# Screening gestational diabetes mellitus: The role of maternal age

**DOI:** 10.1371/journal.pone.0173049

**Published:** 2017-03-15

**Authors:** Chun-Heng Kuo, Szu-Chi Chen, Chi-Tai Fang, Feng-Jung Nien, En-Tzu Wu, Shin-Yu Lin, Lee-Ming Chuang, Chien-Nan Lee, Hung-Yuan Li

**Affiliations:** 1 Department of Internal Medicine, New Taipei City Hospital, New Taipei City, Taiwan; 2 Department of Internal Medicine, National Taiwan University Hospital, Taipei, Taiwan; 3 Institute of Epidemiology and Preventive Medicine, College of Public Health, National Taiwan University, Taipei, Taiwan; 4 Department of Internal Medicine, National Taiwan University Hospital, Yun-lin branch, Yun-lin, Taiwan; 5 Department of Obstetrics and Gynecology, Dianthus MFM Clinic, Taiwan; 6 Department of Obstetrics and Gynecology, National Taiwan University Hospital, Taipei, Taiwan; University of Catanzaro, ITALY

## Abstract

**Objective:**

Using a specific cutoff of fasting plasma glucose (FPG) to screen gestational diabetes mellitus (GDM) can reduce the use of oral glucose tolerance tests (OGTT). Since the prevalence of GDM increases with age, this screening method may not be appropriate in healthcare systems where women become pregnant at older ages. Therefore, we aimed to develop a screening algorithm for GDM that takes maternal age into consideration.

**Methods:**

We included 945 pregnant women without history of GDM who received 75g OGTT to diagnose GDM in 2011. Screening algorithms using FPG with or without age were developed. Another 362 pregnant women were recruited in 2013–2015 as the validation cohort.

**Results:**

Using FPG criteria alone, more GDM diagnoses were missed in women ≥35 years than in women <35 years (13.2% *vs*. 5.8%, p <0.001). Among GDM women ≥35 years, 63.6% had FPG <92 mg/dL (5.1 mmol/L). Use of the algorithm with an “age plus FPG” cutoff could reduce the use of OGTT (OGTT%) from 77.6% to 62.9%, while maintaining good sensitivity (from 91.9% to 90.2%) and specificity (from 100% to 100%). Similar reduction in OGTT% was found in the validation cohort (from 86.4% to 76.8%). In the simulation, if the percentage of women ≥35 years were 40% or more, the screening algorithm with an “age plus FPG” cutoff could further reduce OGTT% by 11.0%-18.8%.

**Conclusions:**

A screening algorithm for GDM that takes maternal age into consideration can reduce the use of OGTT when women become pregnant at older ages.

## Introduction

Gestational diabetes mellitus (GDM) is defined as carbohydrate intolerance during pregnancy that is developed or recognized for the first time [1, 2]. Pregnant women with GDM have a higher risk of adverse perinatal outcomes, including macrosomia, birth trauma, neonatal jaundice, infant respiratory distress syndrome, and hypertensive disorders of pregnancy, and a need of primary cesarean section [3, 4]. In addition, 15–50% of these GDM women develop type 2 diabetes later in life [1]. Furthermore, based on reports from our group and others, children and adolescents with higher birth weight are associated with increased risk of obesity and diabetes [5, 6]. Therefore, we sometimes call GDM “a disease across two generations”.

In 2010, the International Association of the Diabetes and Pregnancy Study Group (IADPSG) proposed a new diagnostic criterion using 75g oral glucose tolerance tests (OGTT) to diagnose GDM. This new criterion was adopted by the World Health Organization, the American Diabetes Association, and many other organizations [7, 8]. In the IADPSG guideline, all pregnant women are suggested to undergo OGTT. Since OGTT are time-consuming for mothers and labor-intensive for the laboratory, some studies report screening methods to reduce the use of OGTT. Among the reports, two studies from the United Arab Emirates and China used fasting plasma glucose (FPG) to decide whether OGTT is needed [9, 10]. This FPG-based screening method reduced the use of OGTT by 50%, and missed only 4.6% to 12.2% of GDM women.

In the study from the United Arab Emirates, the mean age of the study subjects was 28.3 years [9]. In the study from China, the mean age was not given, but we believe that it would not be high, since women often get pregnant at relatively young ages in China [11]. This is different from the situation in many healthcare systems and countries, especially in most developed countries, where women get pregnant at older ages [12–14]. Besides, in non-pregnant adults, diagnosis of diabetes using FPG criteria alone would miss a significant proportion of older subjects with diabetes [15, 16]. These data suggest that a screening strategy for GDM based on an FPG cutoff value may not be appropriate when women become pregnant at an older age. To the best of our knowledge, the role of maternal age in the screening of GDM has not been explored. Therefore, in this study, we aimed to develop a screening algorithm taking maternal age into consideration to improve the performance of the FPG-based screening algorithm.

## Materials and methods

We conducted two cohort studies for this project, including a retrospective cohort study to develop the screening algorithms (the training cohort) and a prospective cohort study to validate the performance of the algorithms (the validation cohort). In the training cohort, all pregnant women, who delivered a singleton child between January 2011 and December 2011 at National Taiwan University Hospital, Taipei, Taiwan, were recruited retrospectively by reviewing medical records [14]. Their medical and gynecological history, and results of physical examination and laboratory tests were recorded. All pregnant women underwent a 75g OGTT at 24–28 weeks to diagnose GDM. Pregnant women with overt diabetes, defined as diabetes diagnosed before pregnancy, were excluded. Written informed consent was obtained from each patient for the agreement of chart review in the training cohort. On the other hand, the validation cohort was recruited prospectively from November, 2013 to March, 2015. A total of 441 pregnant women were contacted and 375 (85%) agreed to participate this study. All pregnant women in this cohort underwent the same procedures as that in the training cohort. Their medical history, findings from physical examination, and results of laboratory tests were recorded. Written informed consent was obtained from each patient before enrollment in the validation cohort. Both studies were reviewed and approved by the Institutional Review Board of National Taiwan University Hospital.

GDM was diagnosed according to the IADPSG criteria [7]. Specifically, the diagnosis was made when any of the following criteria were met: 1). FPG ≥92 mg/dL (5.1 mmol/L); 2). 1-hour plasma glucose during OGTT (1hPG) ≥180 mg/dL (10.0 mmol/L); 3). 2-hour plasma glucose during OGTT (2hPG) ≥153 mg/dL (8.5 mmol/L). GDM with FPG <92 mg/dL (5.1 mmol/L) was defined as 1hPG ≥180 mg/dL (10.0 mmol/L) or 2hPG ≥153 mg/dL (8.5 mmol/L), and FPG <92 mg/dL (5.1 mmol/L). When the diagnosis of GDM was made, the woman will receive treatment for GDM, including lifestyle modification and self- monitoring of blood glucose, with or without medications. Since it is reasonable for pregnant women with a history of GDM to undergo OGTT, we excluded women with a history of GDM from the analyses in this study (women with history of GDM, N = 3 in the training cohort, and N = 13 in the validation cohort). Information on parity, a history of macrosomia, a history of hypertension, a history of pregnancy-induced hypertension and a history of preeclampsia was acquired by physicians. Body height was recorded to the nearest 0.5 cm (0.2 in) and body weight to the nearest 0.1 kg (0.22 lb). BMI was defined as body weight (kilograms) divided by the square of body height (meters). Large for gestational age was defined as a birth weight above the gender-specific 90^th^ percentile for gestational age.

### Statistical analysis

Data were presented as means and standard deviations for continuous variables, and as number and percentages for categorical variables. Student’s *t* tests, Chi-square tests, and Fisher's exact tests were used to identify the differences in clinical characteristics between the GDM and non-GDM groups. Since pregnant women with FPG ≥ 92 mg/dL (5.1 mmol/L) can be diagnosed as GDM by the definition of the IADPSG, logistic regression analyses were performed to identify important risk factors and to estimate their regression coefficients in women with FPG <92 mg/dL (5.1 mmol/L). Variables significantly associated with GDM in univariate logistic regression models were included in multivariate analyses. Model 1 was constructed by forward and backward selection (both selection methods resulted in the same model) and consisted of variables which were independently associated with GDM in women with FPG <92 mg/dL (5.1 mmol/L), including age, BMI and FPG. Model 2 (the full model) included all the variables which were significantly associated with GDM in women with FPG <92 mg/dL (5.1 mmol/L) in univariate analyses. Since the regression coefficients for age in years and FPG in mg/dL were similar in the statistical model composed of age and FPG, “age plus FPG” was used to develop a screening algorithm to exclude GDM in women with FPG < 92 mg/dl (5.1 mmol/L). Receiver operating characteristic (ROC) curves were used to compare the performance of FPG-only and “age plus FPG” to screen GDM in women with FPG <92 mg/dL (5.1 mmol/L). Two algorithms were developed. Algorithm A used FPG to determine the use of OGTT; algorithm B used “age plus FPG” to decide the use of OGTT. In both algorithms, women with FPG ≥92 mg/dL (5.1 mmol/L) was diagnosed as GDM. To search for the cutoffs to exclude GDM, we calculated the performance of each algorithm to diagnose GDM, including sensitivity, specificity and the percentage of women who received OGTT (OGTT%). Since FPG levels in women with FPG <92 mg/dL (5.1mmol/L) in the training cohort ranged from 60 to 91 mg/dL, we calculated the performance of the algorithm A with FPG cutoffs at 60 to 91, with an increment of 1 (i.e. 60, 61, 62, etc.). In algorithm B, since the values of age in years plus FPG in mg/dL in women with FPG <92 mg/dL (5.1 mmol/L) ranged from 90 to 140 in the training cohort, we calculated the performance of the algorithm with cutoffs of age plus FPG from 90 to 140, with an increment of 1, from 90 to 140 (i.e. 90, 91, 92, etc.). In both algorithms, pregnant women were diagnosed as GDM either by FPG ≥92 mg/dL (5.1 mmol/L) or the results of OGTT. Therefore, the false positive rate (FPR) was 0% for both algorithms. By definition, specificity equals to 1 –FPR. So the specificity for both algorithms is always 100%, no matter which cutoffs were chosen. Since the specificity of both algorithms was always 100%, the determination of optimal cutoffs was a tradeoff between sensitivity and OGTT% (S1 Table, see Supporting Information for more details). We determined the optimal cutoffs in each algorithm when the sensitivity was greater than 90% and the OGTT% was the lowest. Validation was performed in the validation cohort by using the algorithms with the optimal cutoffs determined by the data of the training cohort. Then we simulated the relationship between the percentage of pregnant women older than 35 years and OGTT% by these two algorithms using data from the training cohort. For every cutoff to exclude GDM (60 to 91 in algorithm A and 90 to 140 in algorithm B), OGTT% and sensitivity in women younger than 35 years and in women older than 35 years were calculated separately. The percentage of women older than 35 years was simulated in increments of 10%, from 0% to 100% (i.e. 0%, 10%, 20%, etc.), and OGTT% and sensitivity in the whole population were calculated for each percentage of women older than 35 years. The OGTT% in the whole population was calculated by the following formula: percentage of women younger than 35 years*OGTT% in women younger than 35 years + percentage of women older than 35 years* OGTT% in women older than 35 years. The sensitivity in the whole population was calculated by the following formula: (percentage of women younger than 35 years*percentage of women with GDM by both the IADPSG criteria and the algorithm in women younger than 35 years + percentage of women older than 35 years*percentage of women with GDM by both the IADPSG criteria and the algorithm in women older than 35 years) / (percentage of women younger than 35 years*percentage of women with GDM by the IADPSG criteria in women younger than 35 years + percentage of women older than 35 years*percentage of women with GDM by the IADPSG criteria in women older than 35 years). For each percentage of women older than 35 years, the optimal cutoff was determined when the sensitivity in the whole simulated population was above 90% and the OGTT% was the lowest. A two-tailed p-value below 0.05 was considered significant. Statistical analyses were performed using Stata ⁄ SE 11.0 for Windows (StataCorp, College Station, TX, USA).

## Results

A total of 945 pregnant women were included in the training cohort, and another 362 women were included in the validation cohort; in all, 123 (13.0%) women in the training cohort and 38 (10.5%) in the validation cohort were diagnosed as having GDM. In the training cohort, women with GDM were older, had more parity, and a higher body weight, body mass index (BMI), FPG, 1hPG, and 2hPG. A higher percentage of women with GDM had a history of pregnancy induced hypertension (PIH). In the validation cohort, women with GDM were older, and had a higher BMI, FPG, 1hPG, and 2hPG (Table 1).

**Table 1:** Clinical characteristics of women with and without gestational diabetes mellitus by 2-hour, 75g oral glucose tolerance tests in the training cohort and in the validation cohort.

	The training cohort			The validation cohort		
	With GDM	Without GDM	P	With GDM	Without GDM	P
N	123 (13.0%)	822 (87.0%)	NA	38 (10.5%)	324 (89.5%)	NA
Age (years)	35.6 (4.0)	33.3 (3.9)	<0.001	35.6 (4.2)	33.7 (3.9)	0.005
Age ≥35 years (N, %)	77 (62.6%)	295 (35.9%)	<0.001	23 (60.5%)	132 (40.7%)	0.02
Parity (1/2/3/more, %)	44/46/8/2	58/35/6/1	0.008	50/45/3/3	56/35/8/1	0.3
History of macrosomia (N, %)	2 (1.6%)	3 (0.4%)	0.1	0 (0%)	2 (0.6%)	1.0
History of hypertension (N, %)	2 (1.6%)	8 (1.0%)	0.6	1 (2.6%)	3 (0.9%)	0.4
History of PIH (N, %)	2 (1.6%)	1 (0.1%)	0.046	2 (5.3%)	3 (0.9%)	0.1
History of preeclampsia (N, %)	0 (0%)	7 (0.9%)	0.6	1 (2.6%)	4 (1.2%)	0.4
BW (kg)	65.4 (10.1)	62.2 (7.7)	<0.001	70.2 (15.3)	62.1 (8.5)	<0.001
BMI (kg/m^2^)	25.9 (3.9)	24.2 (2.8)	<0.001	27.0 (5.3)	24.1 (3.2)	<0.001
FPG (mg/dL) [mmol/L]	86 (12) [4.8 (0.6)]	77 (5) [4.3 (0.3)]	<0.001	85 (10) [4.7 (0.6)]	78 (5) [4.4 (0.3)]	<0.001
1hPG (mg/dL) [mmol/L]	183 (88) [10.2 (4.9)]	128 (24) [7.1 (1.3)]	<0.001	175 (30) [9.7 (1.7)]	129 (24) [7.1 (1.3)]	<0.001
2hPG (mg/dL) [mmol/L]	153 (28) [8.5 (1.5)]	111 (18) [6.1 (1.0)]	<0.001	159 (18) [8.8 (1.0)]	111 (19) [6.2 (1.1)]	<0.001

Mean (standard deviations) or N (%) were shown.

GDM, gestational diabetes mellitus; NA, not applicable; PIH, pregnancy induced hypertension; BW, body weight at the visit for oral glucose tolerance tests; BMI, body mass index at the visit for oral glucose tolerance tests; FPG, fasting plasma glucose during oral glucose tolerance tests; 1hPG, 1-hour plasma glucose during oral glucose tolerance tests; 2hPG, 2-hour plasma glucose during oral glucose tolerance tests.

Plasma glucose in mg/dL = plasma glucose in mmol/L * 18.

**Fig 1:**
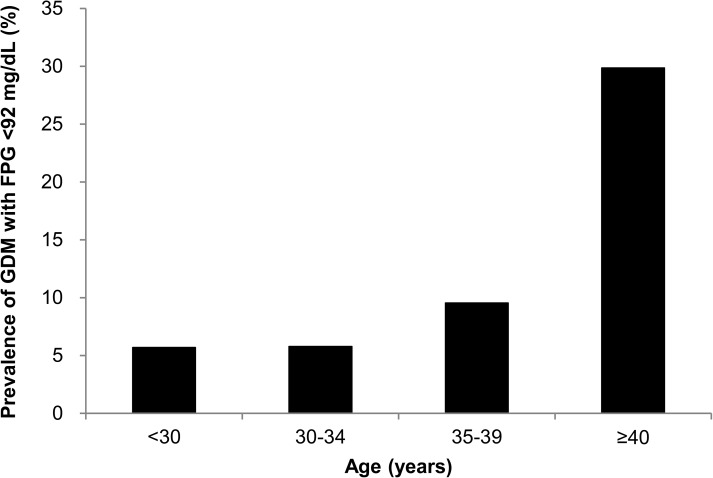
The prevalence of Gestational Diabetes Mellitus (GDM) with Fasting Plasma Glucose (FPG) <92 mg/dL (5.1 mmol/L) in pregnant women by age in the training cohort. Women who have GDM with FPG <92 mg/dL (5.1 mmol/L) will be missed if only FPG is checked. GDM, gestational diabetes mellitus; FPG, fasting plasma glucose.

The prevalence of GDM with FPG <92 mg/dL (5.1 mmol/L) increased with age (Fig 1). The prevalence of GDM with FPG <92 mg/dL (5.1 mmol/L) was 5.7% for the age group below 30 years, 5.8% for the age group 30–34 years, 9.5% for the age group 35–39 years, and 29.9% for the age group above 40 years. In women ≥35 years, the prevalence of GDM with FPG <92 mg/dL (5.1 mmol/L) was significantly higher than that in women younger than 35 years (13.2% *vs*. 5.8%, p <0.001); 63.6% of GDM women ≥35 years had GDM with FPG <92 mg/dL (5.1 mmol/L).

Regression coefficients of risk factors for GDM in women with FPG <92 mg/dL (5.1 mmol/L) are listed in Table 2. Age, parity, history of PIH, BMI, and FPG were significant risk factors for GDM in women with FPG <92 mg/dL (5.1 mmol/L) in univariate analyses. Model 1 consisted of variables which are independently associated with GDM in stepwise multivariate analyses, including age, BMI and FPG. In Model 2 (the full model), age and FPG were significantly associated with GDM in women with FPG <92 mg/dL (5.1 mmol/L), adjusted for parity, history of PIH and BMI. Since BMI usually changes during pregnancy and is not a significant predictor in Model 2, we constructed Model 3 composed of age and FPG only. Both age and FPG were significantly associated with GDM in women with FPG <92 mg/dL (5.1 mmol/L) (Model 3). Since the regression coefficients for age in years and FPG in mg/dL were similar in Model 3, “age plus FPG” was used to develop a screening algorithm for GDM. To compare the performance of “FPG only” and “age plus FPG” cutoffs, ROC curves for a single cutoff to screen GDM in women whose FPG <92 mg/dL (5.1 mmol/L) were shown in S1 Fig (See Supporting Information for more details). OGTT was not included as the screening method. The area under the ROC curve for “age plus FPG” was significantly higher than that for FPG only (0.7145 *vs*. 0.6654, p = 0.0103).

**Table 2:** Regression coefficients (95% confidence interval) of risk factors for gestational diabetes mellitus in women with fasting plasma glucose <92 mg/dL (5.1 mmol/L) in logistic regression models in the training cohort.

	Crude	Model 1	Model 2	Model 3
Age (years)	0.14 (0.09–0.19)***	0.11 (0.05–0.17)***	0.11 (0.05–0.18)***	0.12 (0.06–0.18)***
Parity	0.42 (0.15–0.68)**		0.00 (-0.38–0.38)	
History of PIH (yes vs. no)	2.61 (0.20–5.02)*		1.2 (-1.85–4.18)	
BMI (kg/m2)	0.16 (0.10–0.22)***	0.08 (0.00–0.16)*	0.07 (-0.01–0.16)	
FPG (mg/dL)	0.11 (0.07–0.15)***	0.10 (0.05–0.15)***	0.10 (0.05–0.15)***	0.10 (0.05–0.14)***

* p <0.05.

** p <0.01.

*** p <0.001.

Plasma glucose in mg/dL = plasma glucose in mmol/L * 18.

PIH, pregnancy induced hypertension; BMI, body mass index at the visit for the oral glucose tolerance tests; FPG, fasting plasma glucose.

**Fig 2:**
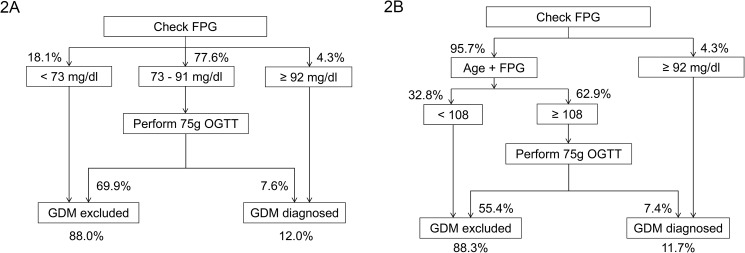
**Algorithm (A) A and (B) B to screen gestational diabetes mellitus.** The percentages of pregnant women in the training cohort are shown. Age + FPG, age in years plus fasting plasma glucose in mg/dL (plasma glucose in mg/dL = plasma glucose in mmol/L * 18). FPG, fasting plasma glucose; GDM, gestational diabetes mellitus; OGTT, oral glucose tolerance tests; GDM excluded, pregnant women who were diagnosed as not having GDM; GDM diagnosed, pregnant women who were diagnosed as GDM.

Then we developed two screening algorithms (Fig 2). Both algorithms used FPG ≥92 mg/dL (5.1 mmol/L) to diagnose GDM. To exclude GDM, algorithm A (Fig 2A) used an FPG cutoff (in mg/dL) and algorithm B (Fig 2B) used an “age plus FPG” cutoff (age in years and FPG in mg/dL). In algorithm A, pregnant women were diagnosed as GDM if their FPG was greater than or equal to 92 mg/dL. If FPG was below 73 mg/dL, GDM was excluded. If FPG was between 73 and 91 mg/dl, OGTT was recommended to confirm the diagnosis of GDM according to the IADPSG criteria. In algorithm B, pregnant women were diagnosed as GDM if their FPG was greater than or equal to 92 mg/dL. Among women with FPG <92 mg/dl, if “age plus FPG” was below 108, GDM was excluded; if “age plus FPG” was greater than or equal to 108, OGTT was suggested to confirm the diagnosis of GDM according to the IADPSG criteria. In Table 3, the OGTT% was reduced from 77.6% in algorithm A to 62.9% in algorithm B in the training cohort. Similarly, the OGTT% was reduced from 86.4% to 76.8% in the validation cohort.

**Table 3:** Performance of algorithm A and B to screen gestational diabetes mellitus in the training cohort and in the validation cohort.

	Cutoff	Threshold to exclude GDM	Training cohort				Validation cohort			
			OGTT (%)	Sensitivity (%)	Specificity (%)	The area under the ROC curve	OGTT (%)	Sensitivity (%)	Specificity (%)	The area under the ROC curve
Algorithm A	FPG	<73	77.6	91.9 (85.6–96)	100	0.959 (0.935–0.984)	86.4	92.1 (78.6–98.3)	100	0.961 (0.917–1)
Algorithm B	“Age plus FPG”	<108	62.9	90.2 (83.6–94.9)	100	0.951 (0.925–0.978)	76.8	92.1 (78.6–98.3)	100	0.961 (0.917–1)

Estimates (95% confidence interval) were shown.

GDM, gestational diabetes mellitus; OGTT, oral glucose tolerance tests; ROC, receiver operating characteristic.

FPG is in conventional units (mg/dL). Plasma glucose in mg/dL = plasma glucose in mmol/L * 18.

The impact of maternal age on OGTT% in both algorithms was evaluated by simulated population with different percentage of pregnant women older than 35 years (percentage35) (S2 Table and S2 Fig, see Supporting Information for more details). In algorithm A, when percentage35 increased from 0% to 100%, the optimal FPG cutoff remained the same, but the OGTT% increased from 75.2% to 81.2%. In algorithm B, the optimal cutoff increased from 103 to 112 when percentage35 increased from 0% to 100%. However, the OGTT% decreased from 78.2% to 63.1% when percentage35 increased from 0% to 40%. When percentage35 was between 40% and 80%, the OGTT% did not decrease further and remained stable between 63.0–62.4%. When percentage35 was above 80%, the OGTT% increased to 65.7% and 70.2%, but the OGTT% by algorithm B was still lower than the OGTT% by algorithm A. Compared to algorithm A, algorithm B reduced more OGTT% with increasing percentage35. When percentage35 was greater than 40%, use of algorithm B resulted in a further reduction of 11.0%-18.8% in OGTT%. This could be explained by the numbers in S2 Table (See Supporting Information for more details). In algorithm B, when percentage35 increased from 0% to 10%, the reduction in OGTT% for women younger than 35 years (12.9%, derived by 78.2%–65.3%) was greater than the increment of OGTT% for women older than 35 years (9.2%, derived by 9.2%–0%). Similar pattern can be found when percentage35 increased from 10% to 40%.

The relationship between pregnancy outcomes and GDM defined by different algorithms was summarized in Table 4 and Table 5. There were 7 subjects whose pregnancy outcomes were not available. The results showed that pregnant women with GDM, compared to those without GDM, had higher percentage of adverse pregnancy outcomes, no matter GDM was defined by algorithm A, algorithm B or the IADPSG criteria (Table 4). In Table 5, GDM defined by algorithm A, algorithm B or the IADPSG criteria was associated with a higher risk of adverse pregnancy outcomes, including Cesarean section, pregnancy-induced hypertension, preeclampsia, preterm delivery and large for gestational age.

**Table 4:** Pregnancy outcomes in women with and without gestational diabetes mellitus defined by algorithm A, B, or the IADPSG criteria.

	Algorithm A		Algorithm B		IADPSG	
	without GDM	with GDM	without GDM	with GDM	without GDM	with GDM
N	827	111	829	109	817	121
Maternal outcomes						
Primary CS (N,%)	173 (21.0%)	29 (26.1%)	174 (21.0%)	28 (25.7%)	172 (21.1%)	30 (24.8%)
CS (N,%)	287 (34.7%)	52 (46.8%)*	286 (34.5%)	53 (48.6%)†	283 (34.6%)	56 (46.3%)*
PIH (N,%)	10 (1.2%)	5 (4.5%)†	10 (1.2%)	5 (4.6%)†	10 (1.2%)	5 (4.1%)*
Preeclampsia (N,%)	9 (1.1%)	6 (5.4%)†	9 (1.1%)	6 (5.5%)†	9 (1.1%)	6 (5.0%)†
Fetal outcomes						
Preterm delivery (N,%)	72 (8.7%)	27 (24.3%)‡	72 (8.7%)	27 (24.8%)‡	71 (8.7%)	28 (23.1%)‡
BW >90^th^ percentile (N,%)	68 (8.2%)	17 (15.3%)*	68 (8.2%)	17 (15.6%)*	68 (8.3%)	17 (14.1%)*
Jaundice (N,%)	185 (22.4%)	30 (27.0%)	184 (22.2%)	31 (28.4%)	182 (22.3%)	33 (27.3%)
Admission to NICU (N,%)	2 (0.2%)	1 (0.9%)	2 (0.2%)	1 (0.9%)	2 (0.2%)	1 (0.8%)
Birth trauma (N,%)	5 (0.6%)	2 (1.8%)	5 (0.6%)	2 (1.8%)	5 (0.6%)	2 (1.7%)
Neonatal hypo- glycemia (N,%)	4 (0.5%)	1 (0.9%)	4 (0.5%)	1 (0.9%)	4 (0.5%)	1 (0.8%)
Fetal death (N,%)	0 (0%)	1 (0.9%)	0 (0%)	1 (0.9%)	0 (0%)	1 (0.8%)

* p<0.05.

† p <0.01.

‡ p <0.001 by chi-squared test, for subjects with or without GDM using different diagnostic algorithms.

GDM, gestational diabetes mellitus; IADPSG, the International Association of the Diabetes and Pregnancy Study Group; CS, Cesarean section; PIH, pregnancy induced hypertension; BW >90^th^ percentile, a birth weight above the gender-specific 90th percentile for gestational age; NICU, neonatal intensive care unit.

**Table 5:** Adjusted odds ratios (95% confidence interval) of gestational diabetes mellitus defined by algorithm A, B, or the IADPSG criteria to predict different pregnancy outcomes.

	Algorithm A	Algorithm B	IADPSG
Maternal outcomes			
Primary CS	1.09 (0.67–1.76)	1.04 (0.64–1.69)	1.01 (0.63–1.61)
CS	1.61 (1.08–2.40)*	1.74 (1.16–2.61)†	1.58 (1.07–2.33)*
PIH	3.85 (1.23–12.05)*	3.93 (1.25–12.32)*	3.47 (1.11–10.87)*
Preeclampsia	5.58 (1.74–17.91)†	5.44 (1.81–16.38)†	4.81 (1.60–14.43)†
Fetal outcomes			
Preterm delivery	3.19 (1.90–5.36)‡	3.25 (1.92–5.48)‡	2.99 (1.80–4.98)‡
BW >90^th^ percentile	2.00 (1.12–3.57)*	1.95 (1.09–3.51)*	1.71 (0.95–3.05)
Jaundice	0.83 (0.51–1.37)	0.89 (0.54–1.46)	0.87 (0.54–1.40)
Admission to NICU	0.09 (0.00–4.47)	0.09 (0.00–4.46)	0.09 (0.00–4.35)
Birth trauma	2.81 (0.51–15.59)	2.87 (0.51–16.03)	2.53 (0.46–14.00)
Neonatal hypoglycemia	0.81 (0.06–10.09)	0.81 (0.06–10.12)	0.74 (0.06–9.16)
Fetal death§	NA	NA	NA

* p<0.05.

† p <0.01.

‡ p <0.001.

Adjusted confounders include gestational week (adjusted for primary Cesarean section [CS], jaundice, admission to NICU, birth trauma, neonatal hypoglycemia, fetal death), parity (adjusted for CS), history of pregnancy-induced hypertension (PIH) and history of preeclampsia (adjusted for PIH and preeclampsia), history of macrosomia (adjusted for BW >90^th^ percentile), maternal age and history of preterm delivery (adjusted for preterm delivery).

§ The only woman with fetal death outcome had GDM defined by either algorithm A, B or the IADPSG criteria.

IADPSG, the International Association of the Diabetes and Pregnancy Study Group; CS, Cesarean section; PIH, pregnancy induced hypertension; BW >90^th^ percentile, a birth weight above the gender-specific 90th percentile for gestational age; NICU, neonatal intensive care unit; NA, not applicable.

## Discussion

In this study, we found, for the first time, that the prevalence of GDM with FPG <92 mg/dL (5.1 mmol/L) increased with age. In other words, if only FPG criterion is used to screen GDM, more women with GDM who become pregnant at an older age will be missed. The algorithm with an FPG cutoff to exclude GDM (algorithm A) could reduce OGTT% by 22.4%. The algorithm with an “age plus FPG” cutoff to exclude GDM (algorithm B) could further reduce the OGTT% by 14.7%. Similar reductions in OGTT% were found in the validation cohort (OGTT%, 86.4% with algorithm A and 76.8% with algorithm B). In the simulation, we found that OGTT% was reduced 11.0%-18.8% more by algorithm B, compared with algorithm A, when there were more than 40% of pregnant women older than 35 years.

In the present study, FPG was a strong predictor for GDM with FPG <92 mg/dL (5.1 mmol/L) and can be used to reduce the use of OGTT. In support of our findings, FPG-based screening methods were shown to reduce OGTT% in studies from the United Arab Emirates [9] and China [10]. In the study from United Arab Emirates, researchers used an FPG cutoff of 79.2 mg/dL to decide the use of OGTT. This method resulted in a reduction of OGTT% to 49.4%, with a high sensitivity of 95.4%. In the study from China, the investigators used the same FPG cutoff. The OGTT% was 49.7%, but the sensitivity was a little bit lower (87.8%). In the present study, the FPG cutoff in algorithm A was lower (73 mg/dL) and the OGTT% was higher (77.6%) than the data reported in the studies from the United Arab Emirates and China. Since the mean age of the study subjects was lower than that in the present study, the higher OGTT% in the present study may result from the different age distribution.

We found that age was a major determinant of GDM with FPG <92 mg/dL (5.1 mmol/L). In support of our findings, several studies have shown that the prevalence of isolated post-load hyperglycemia (IPH) increased with age in non-pregnant adults [15–20]. In the DECODE study, IPH was more prevalent in women than in men in all age groups [21]. The pathogenesis of IPH differs from that of impaired fasting glucose (IFG). Subjects with IFG have marked hepatic insulin resistance with normal or near-normal insulin sensitivity in skeletal muscle and adipose tissue, but subjects with impaired glucose tolerance (IGT) have predominant insulin resistance in skeletal muscle and adipose tissue with only mild hepatic insulin resistance [22–24]. Aging-related glucose intolerance is more prominent in the third decade and continues throughout adulthood [25, 26]. These age-related changes are particularly characterized by an impaired response to glucose challenge, which is partly due to physical inactivity and a decrease in muscle mass [27, 28]. Our findings suggest that age may have a similar effect on insulin resistance in pregnant women, which is worthy of further investigation in the future.

In the present study, the prevalence of GDM with FPG <92 mg/dL (5.1 mmol/L) increased with age, suggesting that the need to use OGTT to diagnose GDM increases for pregnant women with advanced ages. Therefore, we developed algorithm B in this study, which used an “age plus FPG” cutoff. In algorithm B, age is expressed in years and FPG is expressed in mg/dl. For example, a 35-year-old pregnant woman with FPG of 73 mg/dL (4.06 mmol/L) would be recommended to receive a 75g OGTT because 35 plus 73 equals 108. In countries which use SI units, FPG should be converted to conventional unit first. Algorithm B successfully reduced the OGTT% further, compared with algorithm A in both the training and the validation cohorts. The reductions in OGTT% by algorithm B were greater (11.0%-18.8%) when more than 40% of the pregnant women were older than 35 years. In 2013, a report from 11 Mediterranean countries used age, diastolic blood pressure, and FPG to construct a formula to predict the probability of developing GDM [29]. The area under the ROC curve for this formula was good (0.8876), which supports our findings. However, they did not clearly suggest an indication for an OGTT based on the formula, and the formula was complex, so the clinical application was limited. To sum up, when most women become pregnant at a younger age (<35 years), a single FPG cutoff to exclude GDM is recommended and is a simple way to screen GDM. However, with increasing proportion of pregnant women older than 35 years, an “age plus FPG” cutoff to exclude GDM is recommended, especially when more than 40% of pregnant women are older than 35 years.

The strength of this study is that we included two cohorts for the analyses. Both the performance of the algorithms and the effect of age were validated in the validation cohort, which increases the generalizability of the findings. Besides, the algorithms to diagnose GDM proposed in the present study are simple and practical, and can be used clinically. The simulation data further extend the applicability of the algorithms. Third, the FPG cutoffs in the algorithms were searched systemically, but were not decided arbitrarily, which makes the algorithms more reliable. By contrast, there are some limitations of this study. First, since this study enrolled only Han Chinese pregnant women, the results may not be generalizable to other ethnic groups. Indeed, Sacks et al. have reported that the percentage of GDM diagnosed by FPG criteria varied in different centers and countries in the Hyperglycemia and Adverse Pregnancy Outcome (HAPO) study. Further studies in different ethnic groups are warranted to investigate the impact of age on FPG-based screening methods for GDM. Second, we used the IADPSG criteria to diagnose GDM in this study, so the findings may not be applicable to subjects for which different criteria were used to diagnose GDM.

In conclusion, using FPG only to screen GDM, more women with GDM who become pregnant at an older age will be missed. A screening algorithm for GDM taking maternal age into consideration can reduce the use of OGTT when more women become pregnant at an older age.

## Supporting information

S1 Table Performance of algorithm A and B to screen gestational diabetes mellitus using different cutoffs in the training cohort. FPG, fasting plasma glucose; GDM, gestational diabetes mellitus; OGTT, oral glucose tolerance tests; Sen, sensitivity; Spe, specificity; PPV, positive predictive value; NPV, negative predictive value; FPR, false positive rate; FNR, false negative rate.
(DOC)

S2 Table The relationship between different percentage of pregnant women older than 35 years (Percentage 35), cutoffs to exclude gestational diabetes mellitus, and the need of Oral Glucose Tolerance Tests (OGTT%) using algorithms A and B. Percentage 35, percentage of pregnant women older than 35 years; OGTT%, the need of oral glucose tolerance tests; NA, not applicable.
(DOC)

S1 Fig Receiver Operating Characteristic (ROC) curves for Fasting Plasma Glucose (FPG) only or “age plus FPG” to screen gestational diabetes mellitus in women with fasting plasma glucose <92 mg/dL (5.1 mmol/L). Close circles, FPG only, area under the ROC curve = 0.6654 (95% CI = 0.5988–0.7320); hollow squares, “age plus FPG”, area under the ROC curve = 0.7145 (95% CI = 0.6540–0.7750). Comparing the two area under the ROC curves, p = 0.0103.
(TIF)

S2 Fig The relationship between the percentage of pregnant women older than 35 years and the percentage of women who received OGTT (OGTT%) by algorithm A and B. Algorithm A used fasting plasma glucose (FPG) only and did not consider the effect of age; algorithm B used age plus FPG to consider the effect of age. Cutoffs were determined to keep the sensitivity of the whole population greater than 90% with the lowest OGTT%. OGTT, oral glucose tolerance tests.
(TIF)

S1 File The dataset underlying this research. BW, body weight; OGTT, oral glucose tolerance tests; HTN, hypertension; PIH, pregnancy induced hypertension; FPG, fasting plasma glucose; 1hPG, 1-hour plasma glucose during OGTT; 2hPG, 2-hour plasma glucose during OGTT; GDM, gestational diabetes mellitus; IADPSG, the International Association of the Diabetes and Pregnancy Study Group.
(XLSX)

## Abbreviations

BMI: body mass index
FPG: fasting plasma glucose
GCT: glucose challenge tests
GDM: gestational diabetes mellitus
IADPSG: the International Association of the Diabetes and Pregnancy Study Group
HAPO: the Hyperglycemia and Adverse Pregnancy Outcome
IFG: impaired fasting glucose
IGT: impaired glucose tolerance
IPH: isolated post-load hyperglycemia
OGTT: oral glucose tolerance tests
OGTT%: the percentage of women who received OGTT
percentage35: percentage of pregnant women older than 35 years
PIH: pregnancy induced hypertension
ROC: receiver operating characteristic
1hPG: 1-hour plasma glucose during OGTT
2hPG: 2-hour plasma glucose during OGTT

## References

[1] Committee on Practice Bulletins—Obstetrics. Practice Bulletin No. 137: Gestational diabetes mellitus. Obstetrics and gynecology. 2013;122(2 Pt 1):406–16. Epub 2013/08/24. 23969827 10.1097/01.AOG.0000433006.09219.f1

[2] Classification and diagnosis of diabetes mellitus and other categories of glucose intolerance. National Diabetes Data Group. Diabetes. 1979;28(12):1039–57. Epub 1979/12/01. 510803 10.2337/diab.28.12.1039

[3] National Institutes of Health consensus development conference statement: diagnosing gestational diabetes mellitus, March 4–6, 2013. Obstetrics and gynecology. 2013;122(2 Pt 1):358–69. Epub 2013/08/24. 23969806 10.1097/AOG.0b013e31829c3e64

[4] Crowther CA, Hiller JE, Moss JR, McPhee AJ, Jeffries WS, Robinson JS. Effect of treatment of gestational diabetes mellitus on pregnancy outcomes. N Engl J Med. 2005;352(24):2477–86. Epub 2005/06/14. 10.1056/NEJMoa042973 15951574

[5] Wei JN, Li HY, Sung FC, Lin CC, Chiang CC, Li CY, et al Birth weight correlates differently with cardiovascular risk factors in youth. Obesity (Silver Spring, Md). 2007;15(6):1609–16. Epub 2007/06/15. 10.1038/oby.2007.190 17557999

[6] Hillier TA, Pedula KL, Schmidt MM, Mullen JA, Charles MA, Pettitt DJ. Childhood obesity and metabolic imprinting: the ongoing effects of maternal hyperglycemia. Diabetes care. 2007;30(9):2287–92. Epub 2007/05/24. 17519427 10.2337/dc06-2361

[7] Metzger BE, Gabbe SG, Persson B, Buchanan TA, Catalano PA, Damm P, et al International association of diabetes and pregnancy study groups recommendations on the diagnosis and classification of hyperglycemia in pregnancy. Diabetes care. 2010;33(3):676–82. Epub 2010/03/02. PubMed Central PMCID: PMC2827530. 10.2337/dc09-1848 20190296 PMC2827530

[8] McIntyre HD, Colagiuri S, Roglic G, Hod M. Diagnosis of GDM: A suggested consensus. Best Practice & Research Clinical Obstetrics Gynaecology. 2015;29(2):194–205. Epub 2014/09/23. 25242583 10.1016/j.bpobgyn.2014.04.022

[9] Agarwal MM, Dhatt GS, Shah SM. Gestational diabetes mellitus: simplifying the international association of diabetes and pregnancy diagnostic algorithm using fasting plasma glucose. Diabetes care. 2010;33(9):2018–20. Epub 2010/06/04. PubMed Central PMCID: PMC2928355. 10.2337/dc10-0572 20519664 PMC2928355

[10] Zhu WW, Fan L, Yang HX, Kong LY, Su SP, Wang ZL, et al Fasting plasma glucose at 24–28 weeks to screen for gestational diabetes mellitus: new evidence from China. Diabetes care. 2013;36(7):2038–40. Epub 2013/03/29. PubMed Central PMCID: PMC3687275. 10.2337/dc12-2465 23536582 PMC3687275

[11] Xu X, Liu S, Rao Y, Shi Z, Wang L, Sharma M, et al Prevalence and Sociodemographic and Lifestyle Determinants of Anemia during Pregnancy: A Cross-Sectional Study of Pregnant Women in China. International journal of environmental research and public health. 2016;13(9). Epub 2016/09/21. PubMed Central PMCID: PMCPMC5036741. 10.3390/ijerph13090908 PMC5036741 27649213

[12] Astolfi P, Zonta LA. Delayed maternity and risk at delivery. Paediatric and perinatal epidemiology. 2002;16(1):67–72. Epub 2002/02/22. 11856456 10.1046/j.1365-3016.2002.00375.x

[13] Laopaiboon M, Lumbiganon P, Intarut N, Mori R, Ganchimeg T, Vogel JP, et al Advanced maternal age and pregnancy outcomes: a multicountry assessment. BJOG: An International Journal of Obstetrics & Gynaecology. 2014;121 Suppl 1:49–56. 10.1111/1471-0528.12659 24641535

[14] Wu ET, Nien FJ, Kuo CH, Chen SC, Chen KY, Chuang LM, et al Diagnosis of more gestational diabetes lead to better pregnancy outcomes: Comparing the International Association of the Diabetes and Pregnancy Study Group criteria, and the Carpenter and Coustan criteria. Journal of Diabetes Investigation. 2016;7(1):121–6. Epub 2016/01/28. PubMed Central PMCID: PMCPmc4718104. 10.1111/jdi.12378 26816609 PMC4718104

[15] Lai YC, Li HY, Hung CS, Lin MS, Shih SR, Ma WY, et al Performance of homeostasis model assessment and serum high-sensitivity C-reactive protein for prediction of isolated post-load hyperglycaemia. Diabetic Medicine. 2013;30(3):318–25. Epub 2012/09/06. 10.1111/dme.12008 22946586

[16] Resnick HE, Harris MI, Brock DB, Harris TB. American Diabetes Association diabetes diagnostic criteria, advancing age, and cardiovascular disease risk profiles: results from the Third National Health and Nutrition Examination Survey. Diabetes care. 2000;23(2):176–80. Epub 2000/06/27. 10868827 10.2337/diacare.23.2.176

[17] de Pablos-Velasco PL, Martinez-Martin FJ, Rodriguez-Perez F, Ania BJ, Losada A, Betancor P. Prevalence and determinants of diabetes mellitus and glucose intolerance in a Canarian Caucasian population—comparison of the 1997 ADA and the 1985 WHO criteria. The Guia Study. Diabetic Medicine. 2001;18(3):235–41. Epub 2001/04/25. 11318846 10.1046/j.1464-5491.2001.00451.x

[18] Bando Y, Ushiogi Y, Okafuji K, Toya D, Tanaka N, Fujisawa M. The relationship of fasting plasma glucose values and other variables to 2-h postload plasma glucose in Japanese subjects. Diabetes care. 2001;24(7):1156–60. Epub 2001/06/26. 11423495 10.2337/diacare.24.7.1156

[19] Qiao Q, Nakagami T, Tuomilehto J, Borch-Johnsen K, Balkau B, Iwamoto Y, et al Comparison of the fasting and the 2-h glucose criteria for diabetes in different Asian cohorts. Diabetologia. 2000;43(12):1470–5. Epub 2001/01/11. 10.1007/s001250051557 11151755

[20] Hosseinpanah F, Rambod M, Reza Ghaffari HR, Azizi F. Predicting isolated postchallenge hyperglycaemia: a new approach; Tehran Lipid and Glucose Study (TLGS). Diabetic Medicine. 2006;23(9):982–9. Epub 2006/08/23. 10.1111/j.1464-5491.2006.01939.x 16922704

[21] The DECODE Study Group. Age- and sex-specific prevalences of diabetes and impaired glucose regulation in 13 European cohorts. Diabetes care. 2003;26(1):61–9. Epub 2002/12/28. 12502659 10.2337/diacare.26.1.61

[22] Xu L, Jiang CQ, Lam TH, Cheng KK, Yue XJ, Lin JM, et al Impact of impaired fasting glucose and impaired glucose tolerance on arterial stiffness in an older Chinese population: the Guangzhou Biobank Cohort Study-CVD. Metabolism: clinical and experimental. 2010;59(3):367–72. Epub 2009/10/16. 19828159 10.1016/j.metabol.2009.08.004

[23] Li CL, Tsai ST, Chou P. Relative role of insulin resistance and beta-cell dysfunction in the progression to type 2 diabetes—The Kinmen Study. Diabetes research and clinical practice. 2003;59(3):225–32. Epub 2003/02/19. 12590020 10.1016/s0168-8227(02)00249-8

[24] Abdul-Ghani MA, Tripathy D, DeFronzo RA. Contributions of beta-cell dysfunction and insulin resistance to the pathogenesis of impaired glucose tolerance and impaired fasting glucose. Diabetes care. 2006;29(5):1130–9. 10.2337/diacare.2951130 16644654

[25] Andres R. Aging and diabetes. The Medical clinics of North America. 1971;55(4):835–46. Epub 1971/07/01. 5141911 10.1016/s0025-7125(16)32479-8

[26] Jackson RA, Blix PM, Matthews JA, Hamling JB, Din BM, Brown DC, et al Influence of ageing on glucose homeostasis. The Journal of clinical endocrinology and metabolism. 1982;55(5):840–8. Epub 1982/11/01. 10.1210/jcem-55-5-840 6749877

[27] Davidson MB. The effect of aging on carbohydrate metabolism: a review of the English literature and a practical approach to the diagnosis of diabetes mellitus in the elderly. Metabolism: clinical and experimental. 1979;28(6):688–705. Epub 1979/06/01. 377005 10.1016/0026-0495(79)90024-6

[28] Baan CA, Stolk RP, Grobbee DE, Witteman JC, Feskens EJ. Physical activity in elderly subjects with impaired glucose tolerance and newly diagnosed diabetes mellitus. American journal of epidemiology. 1999;149(3):219–27. Epub 1999/02/02. 9927216 10.1093/oxfordjournals.aje.a009795

[29] Savona-Ventura C, Vassallo J, Marre M, Karamanos BG. A composite risk assessment model to screen for gestational diabetes mellitus among Mediterranean women. Internation Journal of Gynaecology and Obstetrics. 2013;120(3):240–4. Epub 2013/01/03. 10.1016/j.ijgo.2012.10.016 23279935

