# Supplementary material for: Screening gestational diabetes mellitus: The role of maternal age
Source: PLoS One. 2017 Mar 15;12(3):e0173049. doi: 10.1371/journal.pone.0173049 (PMC5351872; doi:10.1371/journal.pone.0173049)
Supplement: S1 Table — FPG, fasting plasma glucose; GDM, gestational diabetes mellitus; OGTT, oral glucose tolerance tests; Sen, sensitivity; Spe, specificity; PPV, positive predictive value; NPV, negative predictive value; FPR, false positive rate; FNR, false negative rate. (DOC) [file pone.0173049.s001.doc]

Supplemental Table 1. Performance of algorithm A and B to screen gestational diabetes mellitus using different cutoffs in the training cohort.

| Algorithm A |  |  |  |  |  |  |  |
| --- | --- | --- | --- | --- | --- | --- | --- |
| FPG cutoffs to exclude GDM | OGTT (%) | Sen (%) | Spe (%) | PPV (%) | NPV (%) | FPR (%) | FNR (%) |
| 71 | 85.1 | 95.1 | 100 | 100 | 99.3 | 0 | 4.9 |
| 72 | 81.6 | 93.5 | 100 | 100 | 99.0 | 0 | 6.5 |
| **73** | **77.6** | **91.9** | **100** | **100** | **98.8** | **0** | **8.1** |
| 74 | 71.4 | 89.4 | 100 | 100 | 98.4 | 0 | 10.6 |
| 75 | 65.4 | 87.8 | 100 | 100 | 98.2 | 0 | 12.2 |
| Algorithm B |  |  |  |  |  |  |  |
| “Age plus FPG” cutoffs to exclude GDM | OGTT (%) | Sen (%) | Spe (%) | PPV (%) | NPV (%) | FPR (%) | FNR (%) |
| 106 | 74.0 | 94.3 | 100 | 100 | 99.2 | 0 | 5.7 |
| 107 | 67.9 | 91.1 | 100 | 100 | 98.7 | 0 | 8.9 |
| **108** | **62.9** | **90.2** | **100** | **100** | **98.6** | **0** | **9.8** |
| 109 | 58.7 | 89.4 | 100 | 100 | 98.4 | 0 | 10.6 |
| 110 | 53.0 | 87.8 | 100 | 100 | 98.2 | 0 | 12.2 |

FPG, fasting plasma glucose in mg/dl; GDM, gestational diabetes mellitus; OGTT, oral glucose tolerance tests; Sen, sensitivity; Spe, specificity; PPV, positive predictive value; NPV, negative predictive value; FPR, false positive rate; FNR, false negative rate.
