# Supplementary material for: Screening gestational diabetes mellitus: The role of maternal age
Source: PLoS One. 2017 Mar 15;12(3):e0173049. doi: 10.1371/journal.pone.0173049 (PMC5351872; doi:10.1371/journal.pone.0173049)
Supplement: S2 Table — Percentage 35, percentage of pregnant women older than 35 years; OGTT%, the need of oral glucose tolerance tests; NA, not applicable. (DOC) [file pone.0173049.s002.doc]

**Supplemental Table 2. The relationship between different percentage of pregnant women older than 35 years (Percentage35), cutoffs to exclude gestational diabetes mellitus, and the need of oral glucose tolerance tests (OGTT%)** using algorithms A and B.

| Population |  | <35 years |  | ≥35 years |  | All |  |  |
| --- | --- | --- | --- | --- | --- | --- | --- | --- |
| **Percentage35** | Cutoff* | OGTT% (%) in women <35 years | OGTT% (%) in population† | OGTT% (%) in women ≥35 years | OGTT% (%) in population**‡** | OGTT (%) | Difference in OGTT% (%)§ | Sensitivity (%)‡ |
| **Algorithm A** |  |  |  |  |  |  |  |  |
| **0%** | **73** | **75.2** | **75.2** | **NA** | **0** | **75.2** | **NA** | **91.3** |
| **10%** | **73** | **75.2** | **67.7** | **81.2** | **8.1** | **75.8** | **NA** | **91.5** |
| **20%** | **73** | **75.2** | **60.2** | **81.2** | **16.2** | **76.4** | **NA** | **91.7** |
| **30%** | **73** | **75.2** | **52.7** | **81.2** | **24.4** | **77** | **NA** | **91.8** |
| **40%** | **73** | **75.2** | **45.1** | **81.2** | **32.5** | **77.6** | **NA** | **91.9** |
| **50%** | **73** | **75.2** | **37.6** | **81.2** | **40.6** | **78.2** | **NA** | **92** |
| **60%** | **73** | **75.2** | **30.1** | **81.2** | **48.7** | **78.8** | **NA** | **92** |
| **70%** | **73** | **75.2** | **22.6** | **81.2** | **56.8** | **79.4** | **NA** | **92.1** |
| **80%** | **73** | **75.2** | **15** | **81.2** | **64.9** | **80** | **NA** | **92.1** |
| **90%** | **73** | **75.2** | **7.5** | **81.2** | **73.1** | **80.6** | **NA** | **92.2** |
| **100%** | **73** | **NA** | **0** | **81.2** | **81.2** | **81.2** | **NA** | **92.2** |
| **Algorithm B** |  |  |  |  |  |  |  |  |
| **0%** | **103** | **78.2** | **78.2** | **NA** | **0** | **78.2** | **3** | **93.5** |
| **10%** | **104** | **72.6** | **65.3** | **91.7** | **9.2** | **74.5** | **-1.3** | **91.6** |
| **20%** | **106** | **63.5** | **50.8** | **90.1** | **18** | **68.8** | **-7.6** | **90.7** |
| **30%** | **106** | **63.5** | **44.5** | **90.1** | **27** | **71.5** | **-5.5** | **92.8** |
| **40%** | **108** | **48.3** | **29** | **85.2** | **34.1** | **63.1** | **-14.5** | **90.3** |
| **50%** | **109** | **42.9** | **21.5** | **83.1** | **41.5** | **63** | **-15.2** | **90.8** |
| **60%** | **110** | **35.8** | **14.3** | **79.6** | **47.7** | **62.1** | **-16.7** | **91** |
| **70%** | **111** | **30.5** | **9.2** | **76.1** | **53.3** | **62.4** | **-17** | **91** |
| **80%** | **112** | **25.1** | **5** | **70.2** | **56.1** | **61.2** | **-18.8** | **90.4** |
| **90%** | **112** | **25.1** | **2.5** | **70.2** | **63.1** | **65.7** | **-14.9** | **91.4** |
| **100%** | **112** | **NA** | **0** | **70.2** | **70.2** | **70.2** | **-11** | **92.2** |

**Percentage35, percentage of pregnant women older than 35 years**; OGTT%, the need of oral glucose tolerance tests; NA, not applicable

*** Optimal cutoff for each Percentage35 was determined when the sensitivity in the whole simulated population was above 90% and the OGTT% was the lowest.**

† Calculated by (1 – **Percentage35**)* OGTT% in women <35 years.

‡ Calculated by **Percentage35*** OGTT% in women ≥35 years.

§ vs. algorithm A.

**‡ Calculated by ((1 – Percentage35)* percentage of women with GDM by both the IADPSG criteria and the algorithm in women <35 years + Percentage35* percentage of women with GDM by both the IADPSG criteria and the algorithm in women ≥35 years) / ((1 – Percentage35)* percentage of women with GDM by the IADPSG criteria in women <35 years + Percentage35* percentage of women with GDM by the IADPSG criteria in women ≥35 years).**
