# Supplementary figures and images for: Screening gestational diabetes mellitus: The role of maternal age
Source: PLoS One. 2017 Mar 15;12(3):e0173049. doi: 10.1371/journal.pone.0173049 (PMC5351872; doi:10.1371/journal.pone.0173049)

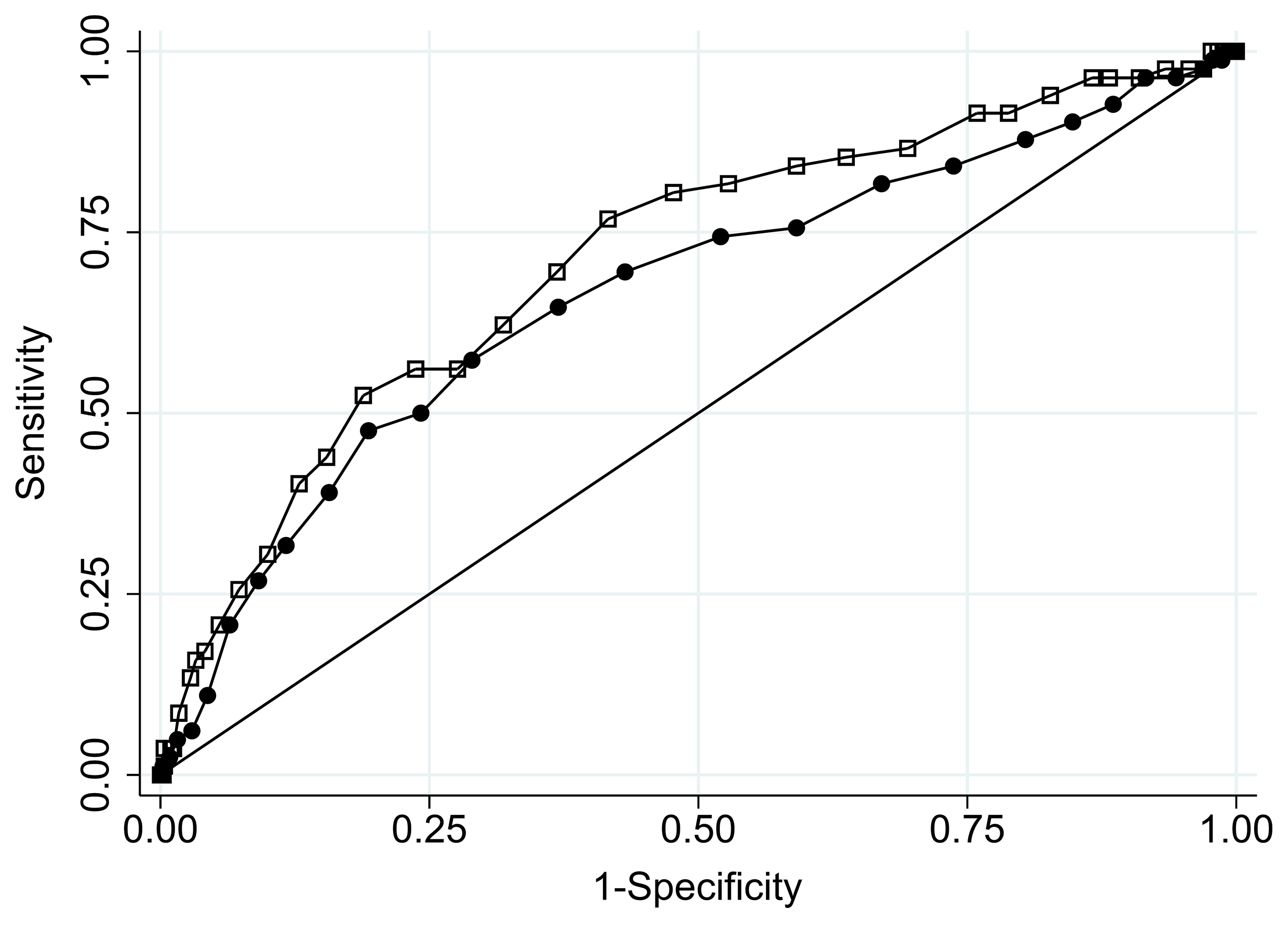

Supplement: S1 Fig — Close circles, FPG only, area under the ROC curve = 0.6654 (95% CI = 0.5988–0.7320); hollow squares, “age plus FPG”, area under the ROC curve = 0.7145 (95% CI = 0.6540–0.7750). Comparing the two area under the ROC curves, p = 0.0103. (TIF) [file pone.0173049.s003.tif]

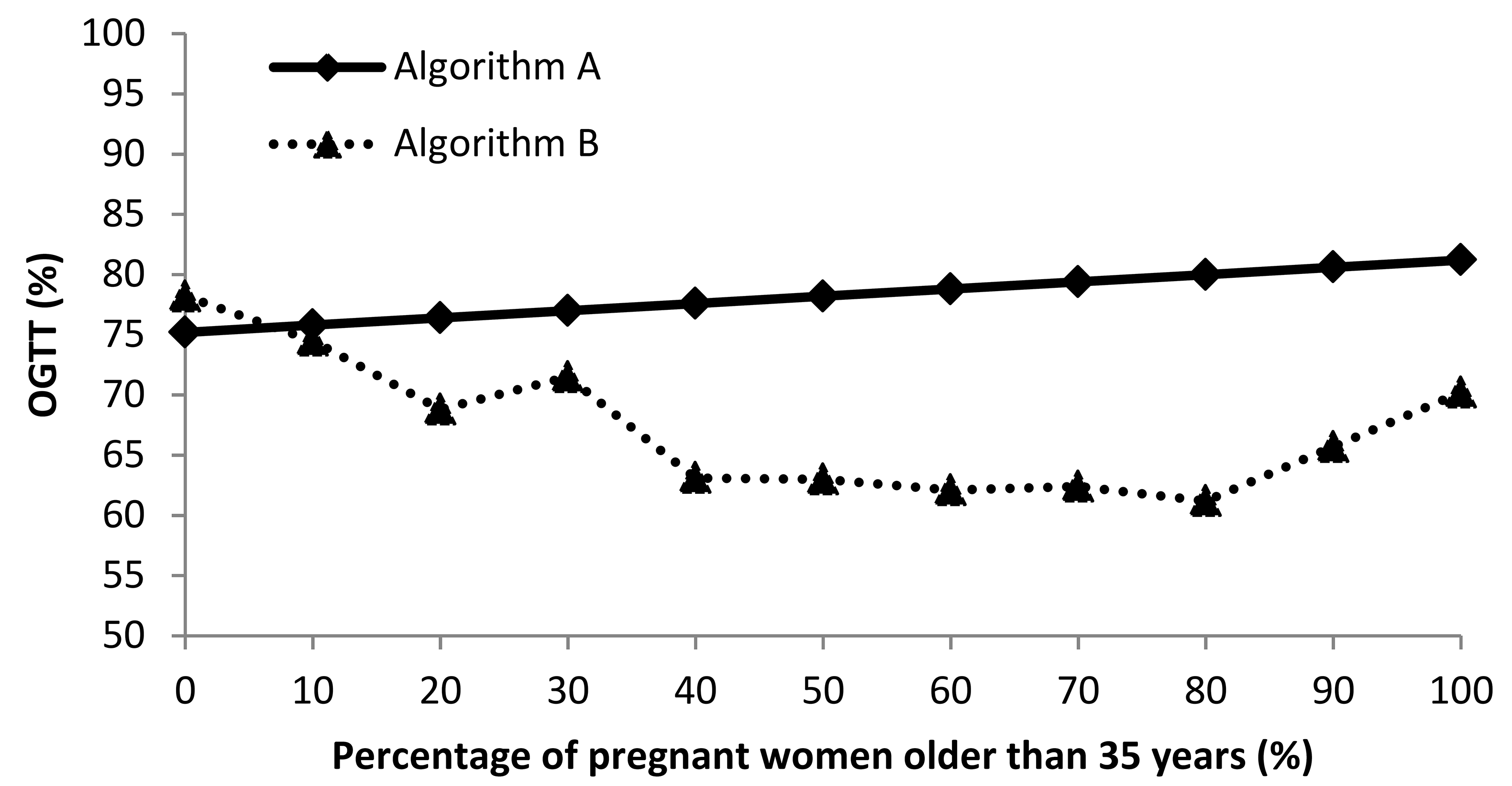

Supplement: S2 Fig — Algorithm A used fasting plasma glucose (FPG) only and did not consider the effect of age; algorithm B used age plus FPG to consider the effect of age. Cutoffs were determined to keep the sensitivity of the whole population greater than 90% with the lowest OGTT%. OGTT, oral glucose tolerance tests. (TIF) [file pone.0173049.s004.tif]
